# The first report of multidrug resistance in gastrointestinal nematodes in goat population in Poland

**DOI:** 10.1186/s12917-020-02501-5

**Published:** 2020-08-03

**Authors:** Marcin Mickiewicz, Michał Czopowicz, Ewelina Kawecka-Grochocka, Agata Moroz, Olga Szaluś-Jordanow, Marián Várady, Alżbeta Königová, Marina Spinu, Paweł Górski, Emilia Bagnicka, Jarosław Kaba

**Affiliations:** 1 Division of Veterinary Epidemiology and Economics, Institute of Veterinary Medicine, Warsaw University of Life Sciences-SGGW, Nowoursynowska 159c, 02-776 Warsaw, Poland; 2 Department of Animal Improvement, Institute of Genetics and Animal Breading, Postępu 36A, Jastrzębiec, 05-555 Magdalenka, Poland; 3 Department of Small Animal Diseases with Clinic, Institute of Veterinary Medicine, Warsaw University of Life Sciences-SGGW, Nowoursynowska 159c, 02-776 Warsaw, Poland; 4 Institute of Parasitology, Slovak Academy of Sciences, Hlinkova 3, 04001 Košice, Slovakia; 5 Department of Infectious Diseases and Preventive Medicine, Law and Ethics, University of Agricultural Sciences and Veterinary Medicine, Calea Mănăștur 3-5, 400372 Cluj-Napoca, Romania; 6 Division of Parasitology and Invasiology, Department of Preclinical Sciences, Institute of Veterinary Medicine, Warsaw University of Life Sciences-SGGW, Ciszewskiego 8, 02-786 Warsaw, Poland

**Keywords:** Anthelmintic resistance, Benzimidazoles, Gastrointestinal nematodes, Goats, Ivermectin, Levamisole

## Abstract

**Background:**

Prophylactic anthelmintic treatment with one of three basic classes of anthelmintics (benzimidazoles, macrocyclic lactones and imidazothiazoles) is still the mainstay of control of gastrointestinal nematode infections in small ruminants worldwide. As a consequence, anthelmintic resistance is a serious threat to small ruminant health and production. While the resistance to one class of anthelmintics has already been reported in most of countries, the newly-emerging problem is the resistance to two or even all of classes referred to as multidrug resistance. This study aimed to evidence the presence of multidrug resistance of gastrointestinal nematodes in goats in Poland.

**Results:**

The combination of one in vivo method (fecal egg count reduction test) and two in vitro methods (egg hatch test and larval development test) performed in two goat herds in the southern Poland showed the presence of gastrointestinal nematodes resistant to fenbendazole and ivermectin in both herds. Moreover, in one herd it revealed the development of resistance to the last effective anthelmintic, levamisole, in response to one-year intensive use. *Haemonchus contortus* was the most prevalent gastrointestinal nematode in samples in which resistance to benzimidazoles and ivermectin was found, whereas *Trichostrongylus colubriformis* predominated when resistance to levamisole was observed.

**Conclusion:**

This study shows for the first time that multidrug resistance of gastrointestinal nematodes to three basic classes of anthelmintics is already present in goat population in Poland. Moreover, it may indicate that different species or genera of gastrointestinal nematodes are responsible for the resistance to specific anthelmintics.

## Background

Parasitic infections, in particular, those caused by gastrointestinal nematodes (GIN), are one of the main factors responsible for economic losses in goat production around the world. The mainstay of GIN control in goats remains the regular use of anthelmintic drugs of three main classes – benzimidazoles, macrocyclic lactones and imidazothiazoles [1, 2]. Nowadays GIN control becomes increasingly problematic due to the emergence of nematodes resistant to one or more classes of anthelmintics. Anthelmintic resistance (AR) has been reported in various GIN in goat herds all over the world, including many European countries. The most prevalent is resistance to benzimidazoles, followed by the resistance to macrocyclic lactones and imidazothiazoles (namely levamisole), with the latter so far only occasionally reported [3]. Anthelmintic resistance distribution appears to correspond to the popularity of anthelmintic classes used in veterinary practice [3]. Over last two decades, simultaneous resistance of goat parasites to more than one anthelmintic class, referred to as multidrug resistance (MDR), has become an increasing problem in Europe, and thus far has been reported in Denmark [4], France [5], Switzerland [6], Slovakia [7] and the United Kingdom [8].

The first cases of anthelmintic resistance to benzimidazoles and macrocyclic lactones have been described only recently in goats in Poland [9, 10]. In this article we describe the first case of MDR in goats to all three basic anthelmintic classes.

## Results

### Fecal egg count reduction tests (FECRT)

The first FECRT carried out in the autumn 2017 showed the resistance to FBZ in both herds irrespective of the calculative method used, and the resistance to IVM in herd A in all three calculative methods while in herd B in calculative method FECR%-1 and FECR%-2. At the same time GIN remained fully susceptible to LEV in both herds. The second FECRT performed after 1 year (autumn 2018) with the use of LEV yielded borderline result in herd A (suspected resistance in method FECR%-1 and lack of resistance in methods FECR%-2 and FECR%-3) and confirmed resistance in herd B according to two of three methods (namely method FECR%-1 and FECR%-2). The last FECRT performed in herd B after a year (autumn 2019) with the use of LEV provided strong evidence of resistance to LEV in all three calculative methods used (Table 1).

**Table 1:** Results of in vivo faecal egg count reduction tests (FECRT) in goat herds A & B

Groups	FECRT no. 1 – autumn 2017				FECRT no. 2 – autumn 2018		FECRT no. 3 – autumn 2019	
	Control	FBZ	IVM	LEV	Control	LEV	Control	LEV
**Herd A**								
n	8	8	8	8	10	10	–	–
FEC [epg] pre-treatment							–	–
mean ± SD	1769 ± 1396	1713 ± 1259	1731 ± 1278	1394 ± 1341	1175 ± 1212	1940 ± 1883		
median (IQR)	1225 (750–2700)	1225 (888–2363)	1225 (950–2338)	1025 (650–1488)	650 (550–1500)	1350 (513–2763)		
range	250–4250	400–3750	450–3700	250–4400	150–4300	250–6400		
FEC [epg] post-treatment^a^							–	–
mean ± SD	1606 ± 1384	1250 ± 810	1188 ± 1144	6 ± 18	815 ± 632	40 ± 97		
median (IQR)	925 (763–2225)	1225 (750–2700)	750 (425–1463)	0 (0–0)	650 (375 − 1163)	0 (0–0)		
range	350–4400	250–4250	200–3350	0–50	50–2100	0–300		
FECR%-1 [%] (CI 95%)	–	22 (−70 to 64) (R)^b^	26 (−89 to 71) (R)	100 (97 to 100) (ND)	–	95 (75 to 99) (S)	–	–
FECR%-2 [%]	–	27 (R)	31 (R)	100 (ND)	–	98 (ND)	–	–
FECR%-3 [%]	–	20 (R)	24 (R)	100 (ND)	–	97 (ND)	–	–
**Herd B**								
n	6	5	5	5	10	10	22	21
FEC [epg] pre-treatment								
mean ± SD	3592 ± 2929	2750 ± 1065	3080 ± 1263	3230 ± 924	4390 ± 2314	6175 ± 3365	2355 ± 1779	2307 ± 1394
median (IQR)	2900 (1038–6000)	3100 (2500–3250)	3250 (2500–3700)	3550 (2700–3800)	3900 (2863–4763)	4825 (4438–6750)	2000 (875–3650)	2100 (1250–3700)
range	900 − 7400	1050–3850	1300–4650	1900–4200	2050–10,050	3500–14,750	200–5950	550–4900
FEC [epg] post-treatment^a^								
mean ± SD	2658 ± 1404	2950 ± 937	250 ± 252	70 ± 157	4980 ± 2376	665 ± 652	1911 ± 1437	1471 ± 1315
median (IQR)	2250 (1863–2825)	3000 (2250–3200)	100 (50–500)	0 (0–0)	4275 (4075–5038)	400 (225–913)	1500 (800–2788)	1350 (600–1950)
range	1400–5300	1950–4350	50–550	0–350	2250–11,000	0–2000	200–5300	0–5300
FECR%-1 [%] (CI 95%)	–	–11 (−88 to 35) (R)	91 (74 to 97) (R)	97 (79 to 100) (ND)	–	87 (73 to 93) (R)	–	23 (−29 to 54) (R)
FECR%-2 [%]	–	–7 (R)	92 (R)	98 (ND)	–	89 (R)	–	36 (R)
FECR%-3 [%]	–	−45 (R)	89 (ND)	97 (ND)	–	91 (ND)	–	21 (R)

*n* number of animals in the group

*FBZ* fenbendazole, *IVM* ivermectin, *LEV* levamisole

*FEC* faecal egg count, *epg* eggs per gram, *FECR%* faecal egg count reduction percentage

^a^ 14 days post-treatment in the autumn 2017, 5–7 days post-treatment in the autumn 2018 and 2019

^b^ anthelmintic resistance: R – confirmed, S – suspected, ND – not detected

In herd A the main GIN detected before the anthelmintic treatment in both FECRT was *Haemonchus contortus* (87–99%), with a very little share of *Teladorsagia circumcincta* (1–8%) and *Trichostrongylus colubriformis* (1–5%; only in the first FECRT). After the treatment only the number of eggs decreased while composition of GIN remained unchanged regardless of the anthelmintic used.

**Fig. 1:**
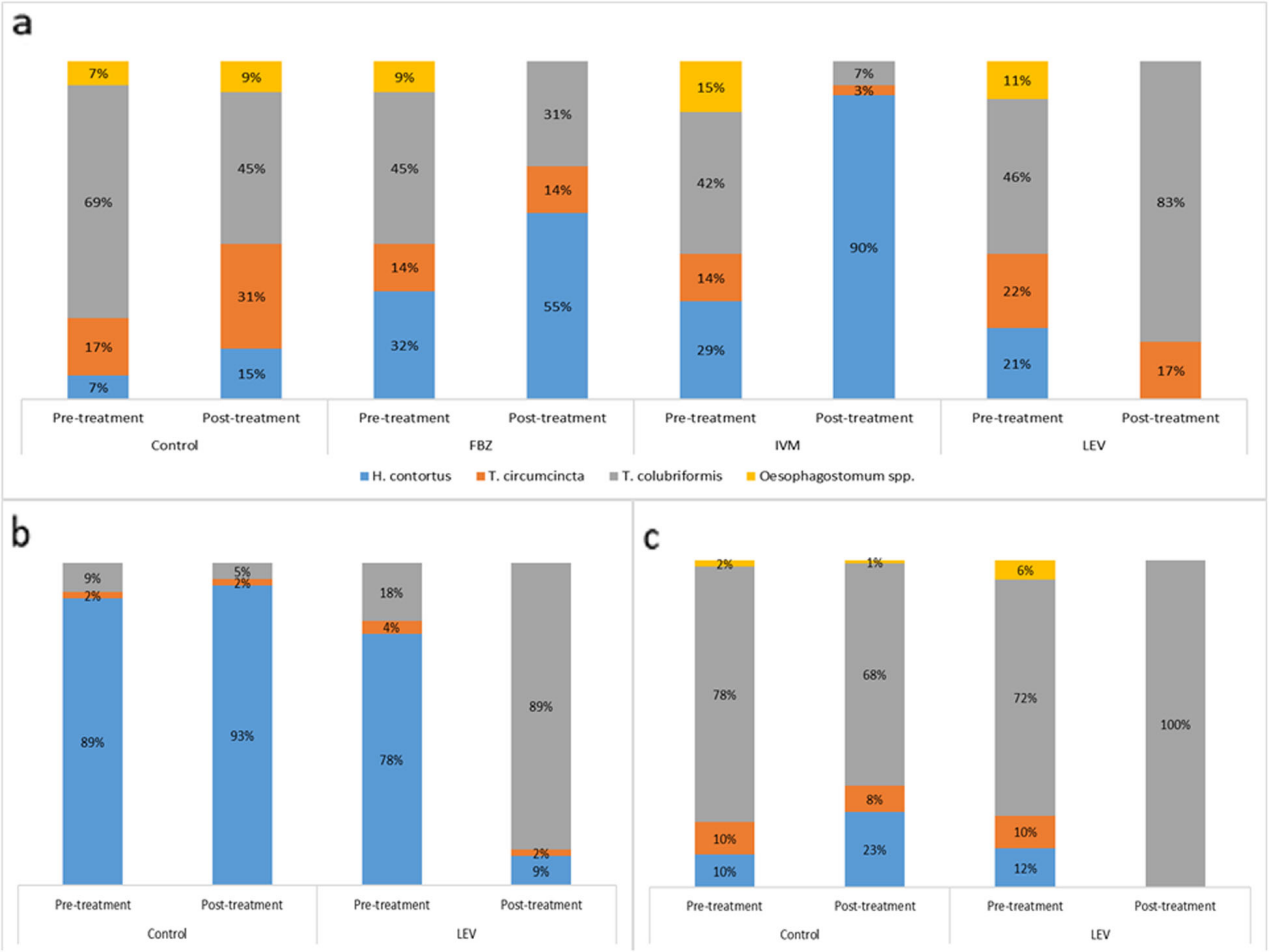
Percentage composition of gastrointestinal nematodes in the first fecal egg count reduction test performed in herd B with the use of fenbendazole (FBZ), ivermectin (IVM) and levamisole (LEV), and of the second and third fecal egg count reduction tests performed in herd B with the use of levamisole alone

In herd B in the autumn 2017 the main GIN was *T. colubriformis*, with the smaller share of *H. contortus* and *T. circumcincta* at nearly the same proportions, and some *Oesophagostomum* spp. Treatment with FBZ did not affect the composition of GIN (except for *Oesophagostomum* spp.), suggesting that resistance to this anthelmintic agent was evenly distributed across GIN. Treatment with IVM reduced the share of *T. circumcincta* and *T. colubriformis* and rendered *H. contortus* the main GIN, indicating its principal role in the resistance to IVM. On the other hand, treatment with LEV selected GIN population for *T. colubriformis*, eliminating most of the other GIN (Fig. 1a). This pattern was even more evident in the second and third FECRT, when *T. colubriformis* became the only GIN detectable in fecal samples (Fig. 1b and c). This indicated that *T. colubriformis* was the main GIN responsible for the resistance to LEV.

### Egg hatch test

Eggs hatching was observed in the wells at and above the established discriminating dose (DD) of TBZ (0.1 μg/ml) in both herds. Percentage of hatching of the eggs at DD was 98.5% (CI 95%: 95.6, 99.5%) in herd A, and 96.4% (CI 95%: 92.8, 98.2%) in herd B, which indicated high resistance in both goat herds (Table 2). ED_50_ was 0.85 (CI 95%: 0.20, 1.49) μg/ml in herd A and 1.08 (CI 95%: 0.44, 1.71) μg/ml in herd B, both values way above the threshold value of 0.1 μg/ml.

**Table 2:** Results of in vitro egg hatch test (EHT) and larval development test (LDT) in goat herds A & B

Anthelmintic agent (DD)	EHT June 2018	LDT June 2018			LDT January 2020		
	TBZ μg/ml (0.10)	TBZ μg/ml (0.08)	IVM ng/ml (21.6)	LEV μg/ml (2.0)	TBZ μg/ml (0.08)	IVM ng/ml (21.6)	LEV μg/ml (2.0)
	Corrected percentage (CI 95%) of						
	hatching eggs at DD	developing infective L3 larvae at DD					
Herd A	98.5 (95.6, 99.5)	85.2 (81.7, 88.1)	99.8 (97.8, 100)	43.2 (36.9, 49.7)	–	–	–
Herd B	96.4 (92.8, 98.2)	87.4 (82.8, 90.9)	93.4 (89.6, 95.9)	62.2 (54.6, 69.3)	27.3 (21.6, 33.9)	56.2 (49.3, 62.9)	85.6 (80.0, 89.8)

*FBZ* fenbendazole, *IVM* ivermectin, *LEV* levamisole, *DD* discriminating dose

### Larval development test

Larval development was observed in all wells. In the first LDT performed in June 2018 percentages of larvae developing at the discriminating dose (DD) indicated high resistance to all anthelmintic agents. In the second LDT performed in January 2020 in herd B percentages of larvae developing at the DD indicated high resistance to IVM-AG and LEV and low resistance to TBZ (Table 2). Compared to the first LDT percentage of larvae developing at DD significantly decreased in terms of TBZ (*p* < 0.001) and IVM-AG (*p* < 0.001), whereas it significantly increased in terms of LEV (*p* < 0.001).

**Fig. 2:**
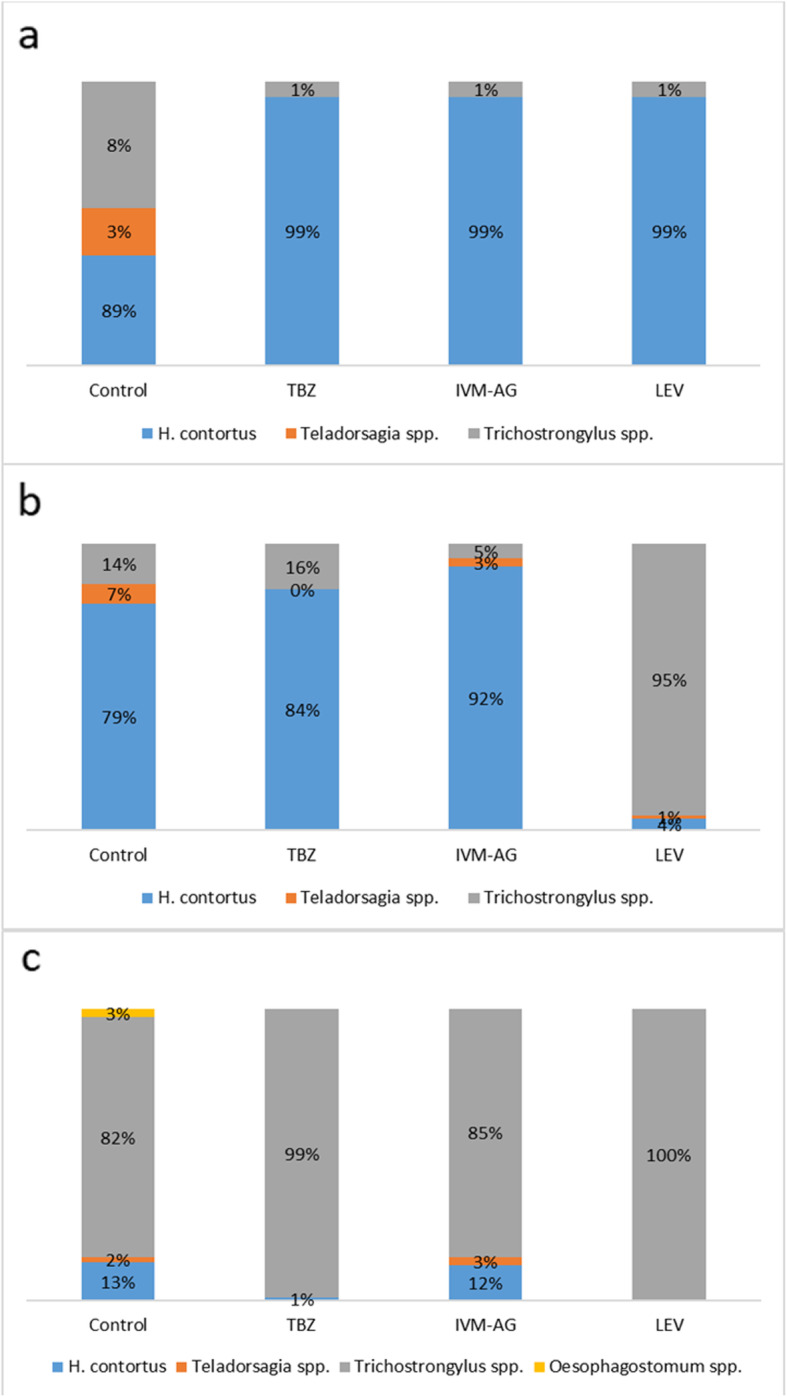
Percentage composition of gastrointestinal nematodes in control wells and in wells containing thiabendazole (TBZ) at discriminating dose of 0.08 μg/ml, ivermectin aglycone (IVM-AG) at discriminating dose of 21.6 ng/ml and levamisole (LEV) at discriminating dose of 2.0 μg/ml in the first larval development test in herd A (**a**) and herd B (**b**), and in the second larval development test in herd B (**c**)

In the first LDT in 2018 in both herds mainly *H. contortus*, with the low proportion of *Trichostrongylus* spp*.* and *Teladorsagia* spp. was found in the control wells*.* In wells containing the three anthelmintics at the DD GIN composition remained unchanged in herd A (Fig. 2a). In herds B GIN composition was also the same in the wells containing TBZ and IVM-AG, while in wells containing LEV *Trichostrongylus* spp*.* predominated (Fig. 2b).

In the second LDT, performed in herd B after more than a year of intensive use of LEV (Additional file 2) *Trichostrongylus* spp*.* remained the main GIN in control wells, with a small share of *H. contortus*, *Teladorsagia* spp*.*, and *Oesophagostomum* spp. In wells with anthelmintics at the DD *Trichostrongylus* spp*.* accounted for virtually all GIN that persisted (Fig. 2c).

### PCR

Identification of GIN based on L3 larvae species-specific features was confirmed by the restriction enzyme cleavage [11].

**Fig. 3:**
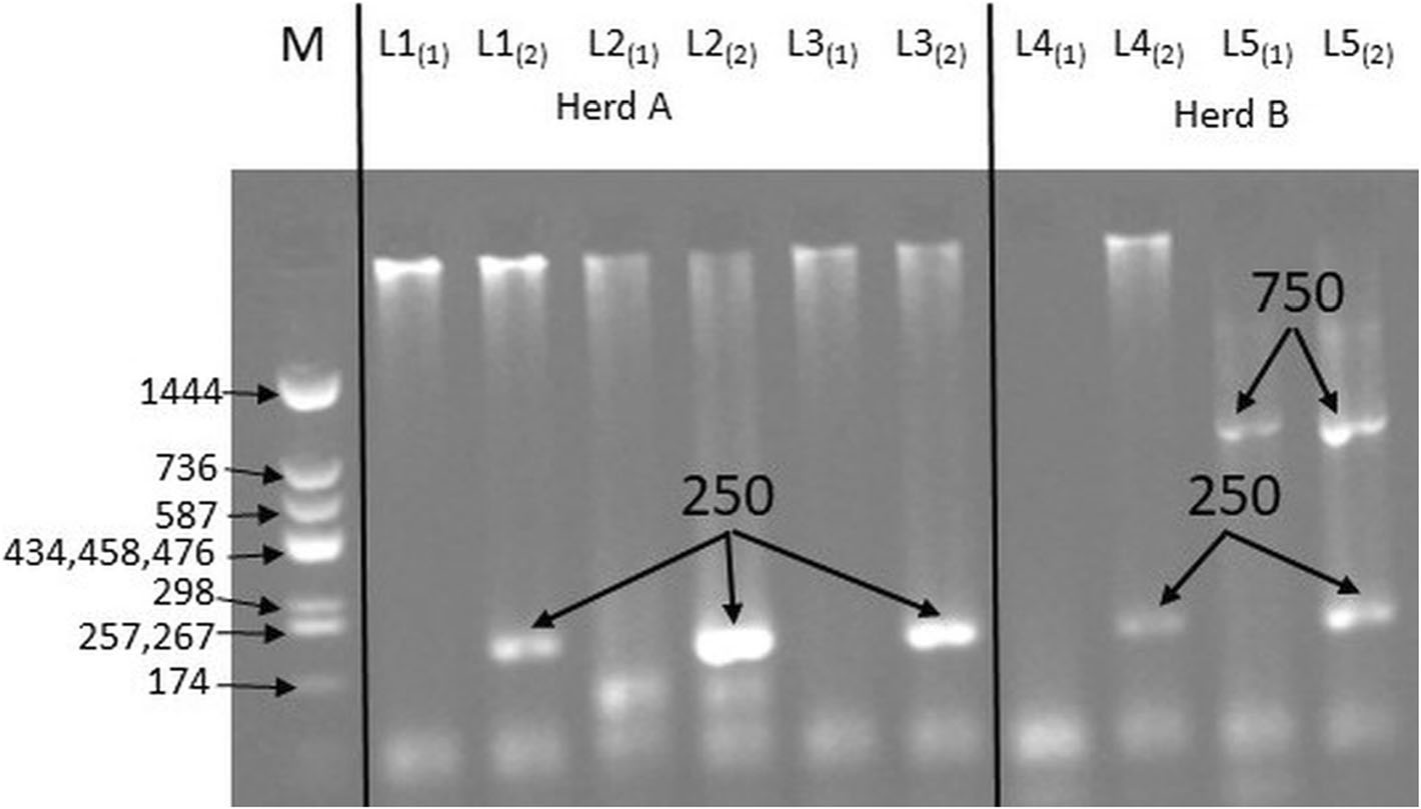
Polymorphism of the length of restriction fragments of variant-1 of the β-tubulin gene indicating the resistance of *Trichostrongylus colubriformis* to benzimidazoles (cropped gel). M – molecular-weight size marker pUC Mix (11–1444) (A&A Biotechnology, Gdańsk, Poland), L1-L5 – presence of gene fragment showing resistance for benzimidazoles (*T. colubriformis*), (1) – the first mixture with three single primers of a given species in ASA-PCR (Pc1 – forward primer, Pc4 – susceptible allele primer, Pc2 – reverse primer), (2) – the second mixture with three single primers of a given species in ASA-PCR (Pc1 – forward primer, Pc3 – resistant allele primer, Pc2 – reverse primer)

**Fig. 4:**
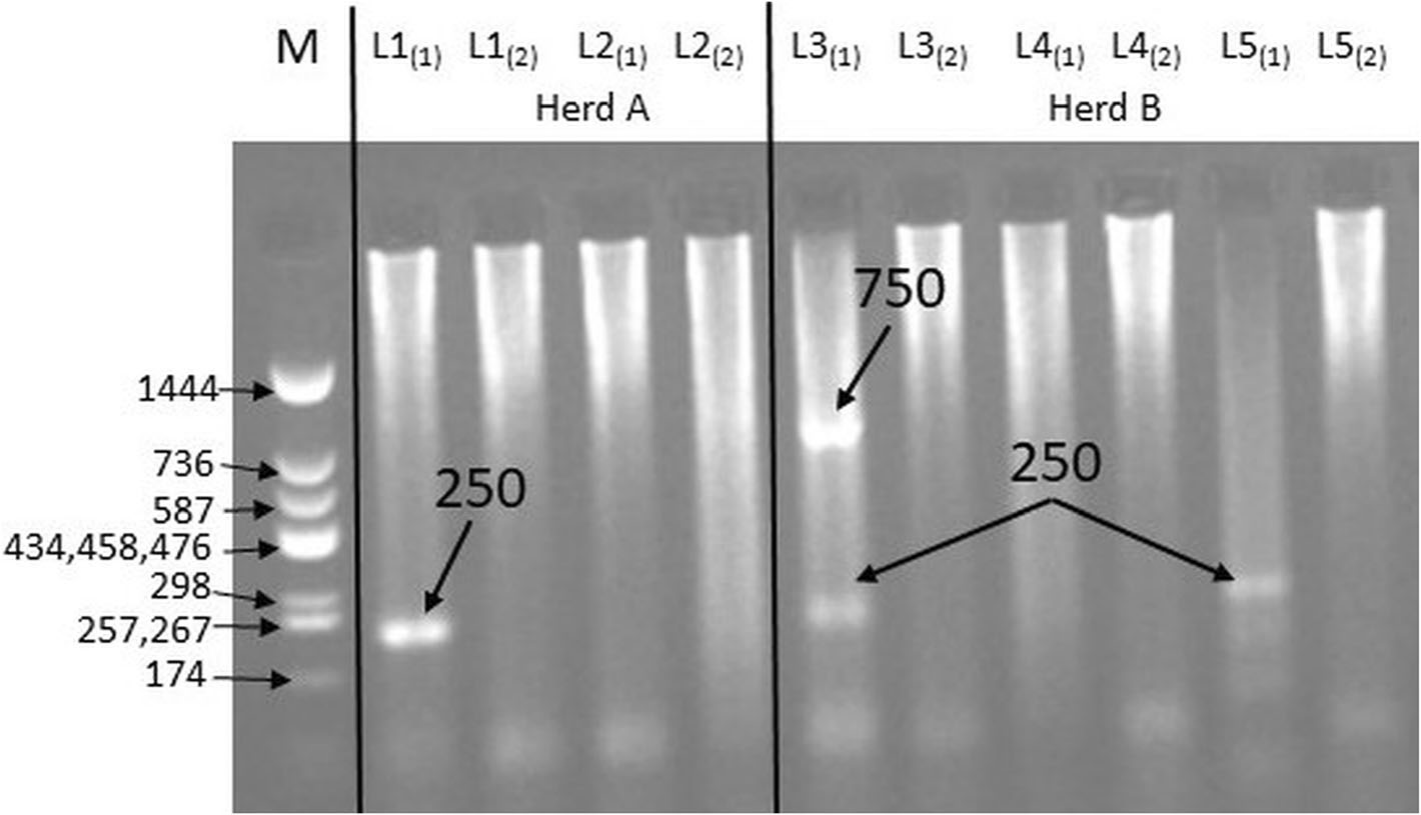
Polymorphism of the length of restriction fragments of variant-1 of the β-tubulin gene indicating the resistance of *Haemonchus contortus* to benzimidazoles (cropped gel). M – molecular-weight size marker pUC Mix (11–1444) (A&A Biotechnology, Gdańsk, Poland), L2, L4 – absence of gene fragment (250 bp) showing resistance for benzimidazoles (*H. contortus*), L1, L3, L5 – presence of gene fragment showing resistance for benzimidazoles (*H. contortus*), (1) – the first mixture with three single primers of a given species in ASA-PCR (Ph1 – forward primer, Ph4 – susceptible allele primer, Ph2 – reverse primer), (2) – the second mixture with three single primers of a given species in ASA-PCR (Ph1 – forward primer, Ph3 – resistant allele primer, Ph2 – reverse primer)

A fragment of the variant of the 1β-tubulin gene (250 bp) associated with resistance of *T. circumcincta* to benzimidazoles was found in goat samples obtained from both herds (Fig. 3, Additional file 4). Moreover, additional fragments were observed which could depend on the geographical origin of the species [12]. Furthermore, fragments of the 1β-tubulin gene showing the resistance of *H. contortus* to benzimidazoles were also found in the samples from both herds (Fig. 4, Additional file 4). The usefulness of the method of separating the primers into two separate mixtures in the case of *T. colubriformis* and *H. contortus,* proposed by Silvestre and Humbert [12], was confirmed. Detection of the gene fragment associated with the resistance to benzimidazoles was possible by using allele-specific PCR. In the control group, after the electrophoretic separation and visualization, no bands of 250 bp were observed, which confirmed that the larvae from the control group were not resistant to benzimidazoles.

## Discussion

This is the first report of resistance to three main classes of anthelmintic drugs in gastrointestinal nematodes of any animal species in Poland. The resistance to benzimidazoles and macrocyclic lactones had been detected first and levamisole remained the only effective anthelmintic in both herds. The second evaluation of levamisole effectiveness performed after a year of its intensive use showed that the resistance to this anthelmintic agent had also emerged.

We used two groups of commonly approved diagnostic methods to confirm AR. The in vivo method was FECRT, whose results were analysed using three different calculative methods [13] – one classical method comparing FEC between treated and control goats [14, 15], another method which evaluated the reduction of FEC only in treated goats [16] and the most complex method including both the change of FEC in time and between the treated and control goats [17]. Resistance to benzimidazoles and macrocyclic lactones was confirmed in both herds regardless of the calculative method used, as was also the resistance to levamisole in herd B. Two in vitro methods were used – EHT for detection of resistance to benzimidazoles, and LDT for detection of resistance to all three anthelmintic classes. The interpretation of these tests’ results depends on the cut-off value (i.e. discriminating dose, DD) used. We adopted cut-off values commonly accepted in parasitological literature. However, given that the DD of levamisole appears to vary between GIN species with 2.5 μg/ml for *H. contortus* [18] and 1.0 μg/ml for *T. colubriformis* [19], results of LDT which we obtained in herd A should be considered as borderline.

In our study we used the combination of the in vivo (FECRT) and the in vitro tests (EHT and LDT), which could increase the sensitivity of the methods for detection of AR in the herds. FECRT is able to detect AR when at least 25% of the population is resistant [20] so its sensitivity is low. Use of FECRT used as the only diagnostic method might result in the underestimation of the AR level on the farms where the resistant alleles are present only in a small proportion of the GIN population. Moreover, the two in vitro tests, EHT and LDT, have the potential to detect a low AR level by using the ED_99_/LC_99_ criterion or a DD [21–23]. Furthermore, the results of Várady et al. [22] also suggest that LDT provides a more accurate quantitative estimate of the level of benzimidazole resistance compared to EHT. In the some cases using the in vitro methods for the detection of AR could be more suitable and sensitive, especially in herds where the resistant alleles are present in the proportion of GIN population [24–26].

The decrease in levamisole effectiveness in herd B was observed after 10 months of exclusive use of this anthelmintic and was first indicated by LDT and then confirmed by FECRT. The quick development of LEV resistance has also been observed in the United States [26], where a decrease in effectiveness occurred after 1 year of extensive use of this anthelmintic. In Europe data on the prevalence of resistance to LEV in goats is limited, however several reports regarding the occurrence of AR to LEV in goat herds have been published in Denmark [4], France [5, 27], and the United Kingdom [28], which may suggest that the resistance to LEV in goats in European countries is still occasional. Our study is the first to reveal the resistance of gastrointestinal nematodes to LEV in Poland. It is interesting that recent reports of goat gastrointestinal nematode resistance to levamisole are from 2009 which may indicate a significant decline in interest in this drug for anthelmintic treatment in goat farms in Europe.

This is the first case of MDR detected in Poland but in our opinion this is just “the tip of the iceberg”. This is mainly due to gaps in farmers’ and veterinarians’ knowledge of factors responsible for the development and spread of AR in ruminant parasites. Goats are known to eliminate various medicines quicker than sheep due to differences in liver metabolism, which results in higher doses needed to ensure anthelmintic efficacy [29, 30]. Extrapolating doses of anthelmintics from cattle or sheep is a common practice in goat herds in Poland. Moreover, lack of anthelmintic agents registered for goats results in frequent repetition of the same treatment [31, 32]. Therefore, our results warrant a complex epidemiological countrywide survey on MDR in Poland.

GIN composition observed in our study implies that different nematode species may be more prone to the development of resistance to particular classes of anthelmintics. *H. contortus* seemed to be the only GIN capable of surviving treatment with IVM, while *T. colubriformis* infection appeared to be the main GIN persisting after the LEV treatment.

Important issue are the origins of MDR in these herds. In both herds a handful of risk factors of AR development were present including high frequency of anthelmintic treatments without anthelmintic class rotation, lack of testing of anthelmintic efficacy, and under-dosing of anthelmintics. Reckless use of anthelmintics appears to be a principal trigger of its development, however, both herds consisted of goats imported from countries in which AR was highly prevalent. Therefore, it is possible that part of GIN with resistant genes had been dragged into these herds along with animals and gave rise to the GIN population with high potential for AR development. Experiences from other countries seem to corroborate this suspicion. The transmission of some resistant parasites between herds via exchange of goats has been described several times in Europe and the United States [6, 23, 33–36]. If this hypothesis was true, goats from these herds would pose a threat to other goats in Poland as a source of multidrug-resistant GIN population. Although, we cannot exclude that the resistant nematodes were dragged into the herds with imported animals, even if it was true, the farmers’ reckless behavior served as a trigger for the development of MDR.

## Conclusions

Concluding, our study shows that MDR of GIN parasites is already present in goat population in Poland. Moreover, it indicates that different species/genera may be responsible for the resistance to specific anthelmintic classes – *H. contortus* appears to be the main GIN resistant to benzimidazoles and ivermectin, and *T. colubriformis* to levamisole. However, this observation needs further investigation and confirmation.

## Methods

### Goat herds

The study was carried out in two related dairy herds (A and B) of pure Anglonubian goats, located in the southern Poland, 275 km apart from each other.

Herd A was established in 2011 by purchasing several adult goats from various locations in Germany, the Netherlands, the United Kingdom and the United States, from which some other goats have been also bought thereafter. Herd B was set up 3 years later by purchasing several adult goats from herd A. In the time period of interest, spanning 4 years between 2017 and 2020, both herds relied on their own replacement. In 2017 herd A counted approximately 40 adult goats and herd B was twofold smaller. Goats in both herds used to be grazed on their own pastures from April to November, 8 h a day. Pasture size was 16 ha and 3 ha, respectively, and they were both grass pastures composed mainly of bluegrass, timothy, darnel, fescue, and clover. Grass was harvested once or twice a year to produce hay and once more in the autumn to produce haylage. Apart for the pasture goats had free access to hay at will, and during milking they were given concentrates made of barley, oat and corn. In the winter they were additionally fed on carrots, fodder beet roots, and haylage. Mineral supplements consisted of mineral blocks with selenium. In herd A goats had a wooden building 110 m^2^ in size, while in the herd B the building was concrete 150 m^2^ in size. In both buildings gravity ventilation was used.

In the spring 2017 in both herds a few adult goats and goat kids died and some goats presented with retarded growth, weight loss and diarrhea. Despite regular and frequent deworming (every 2–4 months on average; Additional files 1 and 2) routine parasitological examinations revealed intensive infection with GIN (strongyle-type eggs). Both farmers were advised to keep goats indoors to prevent environmental exposure to parasitic infections.

### Fecal egg count reduction test (FECRT)

In the autumn 2017 fecal egg counts (FEC) were determined in all adult goats in both herds and they revealed moderate to severe parasitic infection (1128 ± 1234 eggs per gram (epg) and 3183 ± 1721 epg, respectively). On the basis of these results individual goats were selected for the fecal egg count reduction test (FECRT) so that they satisfied the following criteria: age of at least 6 months, pre-treatment FEC of above 150 epg, and no anthelmintic treatment for at least 8 weeks. Thirty six such goats were identified in herd A and 21 in herd B.

FECRT was performed according to the guidelines of the World Association for the Advancement of Veterinary Parasitology (W.A.A.V.P.) described by Coles et al. [14, 15]. Animals were randomly allocated in three treatment groups (*n* = 8 in herd A, *n* = 5 in herd B), and one control group (*n* = 8 in herd A, *n* = 6 in herd B). Prior to the treatment, animals were weighed on an electronic scale. Goats were treated with recommended doses of fenbendazole (FBZ; 10 mg/kg p.o., Fenbenat® oral powder 40 mg/g Vetos-farma, Poland), ivermectin (IVM, 0.3 mg/kg s.c., Biomectin® 10 mg/ml solution for injection, Vetoquinol, Poland), and levamisole (LEV; 12 mg/kg p.o., Levamol® oral powder 80 mg/g, Vetoquinol, Poland) [37]. The medicines were administered by local veterinarians with the owners’ assistance. Oral powders were solved in tap water and given directly into the mouth using a syringe so that the entire dose was consumed by each goat. Control groups were left without any treatment for the time of the study. Fecal samples (at least 10 g of faeces) were collected directly from the rectum on the day of the treatment (day 0), and 14 days after the treatment (day 14). Then, fecal samples were packed in sealed bags, delivered to the laboratory at refrigerator temperature, and examined within 24 h after collection by the modified McMaster technique with an analytical sensitivity of 50 epg according to W.A.A.V.P. guidelines [14, 15]. Larval cultures were prepared for each group by mixing 5 g of faeces collected from each animal on day 0 and on day 14 into one pool per group. After baermannization, a minimum of 100 third-stage larvae (L3) from each pool were identified at the genus/species level following the procedure described by van Wyk and Mayhew [11] and the percentage of each nematode genus/species was calculated. Differentiation between *Trichostrongylus* spp. and *Teladorsagia* spp. was performed after exsheathement of the L3 larvae in 3.5% sodium hypochlorite solution and comparing specific morphological features of GIN species [38].

In July 2018 the second FECRT was performed in both herds to verify LEV efficacy. Twenty goats, aged at least 6 months and with pre-treatment FEC of above 150 epg, were selected accordingly in each herd and divided into the treatment and control group each counting 10 individuals. The further procedures were consistent with the above description for LEV, except for the fact that fecal samples were collected 5 days after LEV administration, so that W.A.V.V.P. recommendations [15], according to which samples for FECRT for LEV should be collected 3 to 7 days after the treatment, were satisfied.

In December 2019 the third FECRT was performed in herd B to verify LEV efficacy. Forty three goats were selected and divided into the treatment and control group counting 21 and 22 individuals, respectively. All the further procedures were the same as in the second FECRT.

### Egg hatch test (EHT)

For in vitro detection of resistance to benzimidazoles egg hatch test (EHT) was performed in both herds in June 2018 at the Institute of Parasitology of the Slovak Academy of Sciences in Kosice according to the method described by Coles et al. [14, 15]. Pooled fresh fecal samples collected from 15 to 20 randomly selected adult goats were homogenized in tap water and used to completely fill 100 ml bottles to ensure anaerobic conditions as described by Hunt and Taylor [39]. Samples were delivered to the laboratory and processed within 24 h after collection. Eggs were extracted from samples by sieving through 250, 100 and 25 μm sieves, centrifugation, and flotation in Sheather’s sugar solution. Then eggs were inspected microscopically to ensure that embryonation had not yet begun, and suspended in deionized water at a concentration of 100 eggs per ml. Eggs suspensions (1.99 ml) were placed in 12 wells of 24-well tissue culture plate (Sarstedt, Germany). A stock solution of thiabendazole (Sigma-Aldrich, Merck, Germany; TBZ) was prepared by dissolving the pure compound in pure dimethyl sulfoxide (SIGMA-ALDRICH, MERCK, Germany; DMSO) according to von Samson-Himmelstjerna et al. [40]. The final concentration was prepared by adding 10 μl of TBZ solution into 1.99 ml of a suspension approximately 100 eggs/ml in water. The final TBZ concentrations used were 0.05, 0.1, 0.3, 0.5 and 1.0 μg/ml. A control (0.5% DMSO) without anthelmintic was also included in the test. The 24-well plates were sealed to prevent drying and incubated for 48 h at 27 °C. The incubation was then terminated by adding 10 μl of Lugol’s iodine to each well. The test was performed with two replicates for each drug concentration. Wells were examined microscopically in inverted microscope (Leica DMi1) at 100x magnification and the number of unhatched gastrointestinal eggs and first-stage larvae (L1) in each well were counted and corrected for natural mortality from control wells (corrected percentage inhibition, cPI). The threshold discriminating dose (DD) of TBZ set at 0.1 μg/ml [14].

### Larval development test (LDT)

The larval development (LDT) test was performed in both herds in June 2018 at the Institute of Parasitology of the Slovak Academy of Sciences in Kosice and in herd B in January 2020 at the laboratory of the Division of Veterinary Epidemiology and Economics, Warsaw University of Life Sciences, Poland. Pooled fresh fecal samples were collected from randomly selected 15–20 adult goats from each herd. Storage and extraction of eggs was the same as described above for EHT. Stock solutions of TBZ (Sigma-Aldrich, Merck, Germany), LEV (Sigma-Aldrich, Merck, Germany) and ivermectin aglycone (Tebu-bio, France; IVM-AG) were prepared by dissolving pure drugs in DMSO (Sigma-aldrich, Merck, Germany) and serially diluted 1:2 in DMSO (TBZ, IVM-AG) or in deionized water (LEV) to produce 12 final concentrations ranging from 0.0006 to 1.28 μg/ml for TBZ, from 0.084 to 173.6 ng/ml for IVM-AG, and from 0.02 to 32 μg/ml for LEV. As it has been demonstrated by Dolinská et al. [21, 41] the use of IVM-AG significantly increased the capacity of the test to distinguish between *H. contortus* strains resistant and susceptible to IVM. Tests were performed according to the procedure described by Hubert and Kerboeuf [42] with further modifications of Várady et al. [43]. Tests were performed in 96 wells cell culture plates (Sarstedt, Germany) with culture medium (150 μl) which consisted of: 10 μl of either TBZ, IVM-AG, LEV or DMSO (control wells) solution, 110 μl of deionised water, 20 μl of culture medium as described by Hubert and Kerboeuf [44] and 10 μl of a suspension (approximately 70–100 eggs) containing amphotericin B (Sigma-Aldrich, Merck, Germany) at a concentration of 5 μg/ml, all in one well of the test plate. Tests were performed using 2 replicates at each drug concentration. The plates were sealed to prevent drying and incubated for 7 days at 27 °C. After the incubation period, 10 μl od Lugol’s solution was added to each well to stop larval development. The unhatched eggs and L1-L3 larvae in each well were counted in an inverted microscope (Leica DMi1 and Olympus CKX53). The L3 larvae in the tested and control wells were identified at the genus/species level following the procedure described by van Wyk and Mayhew [11]. DD for LDT were as follows: TBZ – 0.08 μg/ml [45], IVM-AG – 21.6 ng/ml [21] and LEV – 2 μg/ml [46]. They were higher than traditionally suggested [15] to avoid misclassification of susceptible GIN.

### PCR

#### Extraction of DNA

The L3 larvae obtained from the fecal culture prepared from both herds were used for PCR evaluation. Furthermore, as a control group, L3 larvae obtained from the another herd with no evidence of resistance to benzimidazoles in EHT and LDT were used.

In order to obtain genetic material, the sheath of the larvae was stripped by incubation for 5 min in a Petri dish containing 4 ml larvae suspension and 180 ml sodium hypochlorite (aqueous solution, about 3.5% active Cl, Rectapur1, Prolabo, Singapore). For further analysis, they were diluted in deionized water and stored at − 20 °C. The DNA was isolated using commercial Kit Zymo Research-Quick-DNA™ Microprep Plus Kit (Irvine, USA) according to the manufacturer’s protocol. The quality of DNA was checked by the spectrophotometric NanoDrop1000 (NanoDrop, Waltham, USA) instrument. The absorbance measurements were carried out in 2 μl of the sample at 260/280 nm and 260/230 nm. For further analysis, only samples with a purity of 1.7–2.0 were used.

Nested polymerase chain reaction (nested PCR) with restriction fragment length polymorphism (PCR-RFLP) was performed according to the method given by Silvestre and Humbert [12], with modifications resulting from the specificity of the conducted study (thermal profile and a number of cycles used in the PCR).

The two PCRs were performed using primers designed by Silvestre and Humbert [12] on the basis of the first variant of the β-tubulin gene of two species *T. circumcinta* and *H. contortus* (Additional file 3). In the first PCR the 20 μl reaction mixture contained 2 μl DNA and 10 μl Amplitaq Gold® 360 Master Mix (Applied Biosystems, Life Technologies, USA), 1 μl Pn1 primer, 1 μl Pn2 primer, and 6 μl H_2_O. PCR was performed in a Takara thermocycler (Kusatsu, Japan) using a thermal profile according to the protocol attached to the Amplitaq Gold® 360 Master Mix polymerase. The resulting mixture was used as a DNA template for the next PCR. In the second PCR the 20 μl reaction mixture contained 2 μl of the mixture obtained in the first PCR, 1 μl of Pn3 and Pn4 primers, while the remaining components of the mixture were identical as in the first reaction. The thermal profile was also consistent with the protocol included with the Amplitaq Gold® 360 Master Mix polymerase (Applied Biosystems, Life Technologies, USA). The final product of the nested PCR was stored at − 20 °C to use in the further analyses. The restriction fragment length polymorphism (RFLP) method was used to identify parasitic species. The product obtained in nested PCR was subjected to the activity of the *RsaI* restriction enzyme (Nzytech, Lisbon, Portugal) according to the protocol with the cleavage site: 5′ … GT^AC … 3′.

#### Allele-specific polymerase chain reaction (ASA-PCR)

In the allele-specific PCR, the mixture obtained in nested PCR was used. In the case of *T. circumcinta*, allele-specific products were amplified using two pairs of primers during one PCR. Two of them showed high complementarity (specific primers), while the other two showed low complementarity to the amplified gene fragments (nonspecific primers). In the case of *H. contortus* and *T. colubriformis*, two separate PCRs were performed using 3 primers in one mixture (Additional file 3). The reaction mixture (25 μl) for the amplification of variant 1 fragment of the β-tubulin gene *T. circumcincta* contained 10 μl of Amplitaq Gold® 360 Master Mix (Applied Biosystems, Life Technologies, USA), 4 primers – each 1 μl of Pt1, Pt2, Pt3 and Pt4, 9 μl H_2_O, and 2 μl DNA template obtained as a result of the second nested PCR. Mixtures for PCR with DNA of *H. contortus* and *T. colubriformis* as two separated mixes (both with three primers) were prepared (Additional file 3). PCR was performed for 35 cycles and the amplification temperature was 60 °C. The obtained fragments of the gene were separated by electrophoresis in a 2% agarose gel for 1 hour (55 V, 250A) and then visualized using a GBOX device (Syngene, Bangalore, India). The molecular-weight size marker pUC Mix (11–1444) (A&A Biotechnology, Gdańsk, Poland) was used. Moreover, both nested and allele-specific PCR were performed for the control group of the GIN larvae showing no AR. Original, full-length gel images showing results of PCR for the length of restriction fragments of variant-1 of the β-tubulin gene indicating the resistance of *T. colubriformis* and *H. contortus* to benzimidazoles are presented in Additional file 4.

#### Data analysis

Fecal egg count reduction percentage (FECR%) was calculated using three different methods:

1. Method FECR%-1 = 100% × (1 –T1/C1), according to Coles et al. [14, 15]
2. Method FECR%-2 = 100% × (1 – T1/T0), according to Kochapakdee et al. [16].
3. Method FECR%-3 = 100% × (1 − [T1/T0] × [C0/C1]) according to Dash et al. [17].

T0 and T1 signified the arithmetic mean of FEC (epg) in the treated group on the day of anthelmintic administration and on the last day of the study, respectively. C0 and C1 signified the arithmetic mean of FEC (epg) on the same days in the control group.

In the method FECR%-1 AR was considered present when FECR% was less than 95% and the lower limit of the CI 95% was less than 90%. If only one of these conditions was fulfilled AR was suspected. In the method FECR%-2 AR was indicated by FECR% of less than 95% and in the method FECR%-3 by FECR% of less than 80%.

Results of EHT and LDT were presented as the percentage of hatching eggs or developing L3 stage infective larvae at DD, respectively. AR was defined as hatching of any eggs (in EHT) or development of any stage L3 infective larvae (in LDT) at the DD of a given anthelmintic. AR was numerically expressed as the percentage of eggs/larvae hatching/developing at DD corrected by the percentage hatching/developing in control wells and was classified as low (< 30% development) and high (≥30% development) [21]. Moreover, TBZ concentrations in EHT were log transformed and the S-shaped dose-response curve was fitted by transforming cPI to their probits, defined as normal equivalent deviates (area under the standard normal curve to the left from the position on the curve corresponding to the probability equal to a given cPI) increased by 5 to avoid calculating with negative numbers [47]. The log-probit transformation was used to determine TBZ concentration which inhibits hatching of 50% of eggs (effective dose, ED_50_) [48]. Benzimidazole resistance was considered as confirmed if the ED_50_ value was above 0.1 μg/ml [14].

FEC were presented as the arithmetic mean (±standard deviation, SD), median (interquartile range, IQR), and range. Percentages were compared between groups with the Pearson chi-square test and 95% confidence intervals (CI 95%) were calculated using the Wilson score method [49]. Statistical analysis was performed in TIBCO Statistica 13.3.0 (TIBCO Software Inc., Palo Alto, CA).

## Supplementary information

**Additional file 1.** Anthelmintic treatment used in the herd A in years 2014–2019. Detailed data regarding deworming of goats in the herd A during a 5-year period.

**Additional file 2.** Anthelmintic treatment used in the herd B in years 2014–2019. Detailed data regarding deworming of goats in the herd B during a 5-year period.

**Additional file 3.** The sequences of the primers used in the individual polymerase chain reaction mixtures, the length of the product in base pairs, and the access number in the GenBank database. Detailed data regarding primers used in PCR.

**Additional file 4. **Original, full-length gel images presenting results of PCR for the length of restriction fragments of variant-1 of the β-tubulin gene indicating the resistance of *Trichostrongylus colubriformis* (A) and *Haemonchus contortus* (B) to benzimidazoles. Uncropped gels shown in Fig. 3 (A – *Trichostrongylus colubriformis*) and Fig. 4 (*Haemonchus contortus* – B).

## Data Availability

The data sets used and/or analyzed are available from the corresponding author on reasonable request.

## Abbreviations

AR: Anthelmintic resistance
ASA-PCR: Allele-specific polymerase chain reaction
CI 95%: Confidence interval for the level of confidence of 95%
cPI: Corrected percentage inhibition
DD: Discriminating dose
DMSO: Dimethyl sulfoxide
ED_50_: Effective dose
EHT: Egg hatch test
epg: Eggs per gram
FBZ: Fenbendazole
FEC: Fecal egg count
FECR: Fecal egg count reduction
FECR%: Fecal egg count reduction percentage
FECRT: Fecal egg count reduction test
GIN: Gastrointestinal nematodes
IVM: Ivermectin
IVM-AG: Ivermectin aglycone
LDT: Larval development test
LEV: Levamisole
MDR: Multidrug resistance
PCR: Polymerase chain reaction
PCR-RFPL: Polymerase chain reaction with restriction fragment length polymorphism
RFLP: Restriction fragment length polymorphism
TBZ: Thiabendazole
