# Supplementary material for: The first report of multidrug resistance in gastrointestinal nematodes in goat population in Poland
Source: BMC Vet Res. 2020 Aug 3;16:270. doi: 10.1186/s12917-020-02501-5 (PMC7398340; doi:10.1186/s12917-020-02501-5)
Supplement: Supplementary file 1 — Additional file 1. Anthelmintic treatment used in the herd A in years 2014–2019. Detailed data regarding deworming of goats in the herd A during a 5-year period. [file 12917_2020_2501_MOESM1_ESM.docx]

Additional file 1. Anthelmintic treatment used in the herd A in years 2014-2019

| **Date** | **Anthelmintic** | **Dosage** |
| --- | --- | --- |
| Jan 2016 | eprinomectin | 25 mg/goat spot on |
| Apr 2016 | eprinomectin | 25 mg/goat spot on |
| Jun 2016 | albendazole | 20 mg/kg p.o. |
| Sep 2016 | albendazole | 20 mg/kg p.o. |
| Jan 2017  (for 3 consecutive days) | albendazole | 20 mg/kg p.o. |
| Feb 2017 | albendazole | 20 mg/kg p.o. |
| Mar 2017 | albendazole | 20 mg/kg p.o. |
| Apr 2017 | albendazole | 20 mg/kg p.o. |
| May 2017 | eprinomectin | 50 mg/goat spot on |
| May 2017 | eprinomectin | 1 mg/kg spot on |
| Jun 2017 | eprinomectin | 30 mg/goat spot on |
| Jul 2017 | eprinomectin | 1 mg/kg spot on |
| Jul 2017 | oxfendazole | 3624 mg/female goat, 4530 mg/male goat, 960/1980mg/kids (depending on age) p.o. |
| Jul 2017 | oxfendazole | 3624 mg/female goat, 4530 mg/male goat, 960/1980mg/kids (depending on age) p.o. |
| Jul 2017 | oxfendazole | 3624 mg/female goat, 4530 mg/male goat, 960/1980 mg/kids (depending on age) p.o. |
| Sep 2017 | levamisole ivermectin fenbendazole (FECRT^a^) | 12 mg/kg p.o.  0.3 mg/kg s.c.  20 mg/kg p.o. |
| Oct 2017 | levamisole | 12 mg/kg p.o. |
| Jan 2018 | levamisole | 12 mg/kg p.o. |
| Jul 2018 | levamisole (FECRT^a^) | 12 mg/kg p.o. |

^a^FECRT – fecal egg count reduction test; p.o. – *per os*; s.c. – subcutaneously
