# Supplementary material for: The first report of multidrug resistance in gastrointestinal nematodes in goat population in Poland
Source: BMC Vet Res. 2020 Aug 3;16:270. doi: 10.1186/s12917-020-02501-5 (PMC7398340; doi:10.1186/s12917-020-02501-5)
Supplement: Supplementary file 2 — Additional file 2. Anthelmintic treatment used in the herd B in years 2014–2019. Detailed data regarding deworming of goats in the herd B during a 5-year period. [file 12917_2020_2501_MOESM2_ESM.docx]

Additional file 2. Anthelmintic treatment used in the herd B in years 2014-2019

| **Date** | **Anthelmintic** | **Dosage** |
| --- | --- | --- |
| Apr 2014 | levamisole | 12 mg/kg p.o. |
| Mar 2015 | albendazole | 5 mg/kg p.o. |
| Oct 2015 | albendazole | 5 mg/kg p.o. |
| Apr 2016 | albendazole | 5 mg/kg p.o. |
| Jun 2016 | eprinomectin | 25 mg/goat spot on |
| Aug 2016 | eprinomectin | 25 mg/goat spot on |
| Oct 2016 | albendazole | 5 mg/kg p.o. |
| Jan 2017 | eprinomectin | 25 mg/goat spot on |
| Apr 2017 | albendazole | 5 mg/kg p.o. |
| Jul 2017 | fenbendazole | 10 mg/kg p.o. |
| Oct 2017  FECR^a^ test | levamisole  ivermectin  fenbendazole | 12 mg/kg p.o.  0.3 mg/kg s.c.  20 mg/kg p.o. |
| Nov 2017 | levamisole | 12 mg/kg p.o. |
| Jan 2018 | levamisole | 12 mg/kg p.o. |
| Apr 2018 | levamisole | 12 mg/kg p.o. |
| Jul 2018  FECR^a^ test | levamisole | 12 mg/kg p.o. |
| Aug 2018 | levamisole | 12 mg/kg p.o. |
| Dec 2018 | levamisole | 12 mg/kg p.o. |
| Apr 2019 | levamisole | 12 mg/kg p.o. |
| Dec 2019  FECRT^a^ | levamisole | 12 mg/kg p.o. |

^a^FECRT – fecal egg count reduction test; p.o. – *per os*; s.c. – subcutaneously
