# Supplementary material for: The first report of multidrug resistance in gastrointestinal nematodes in goat population in Poland
Source: BMC Vet Res. 2020 Aug 3;16:270. doi: 10.1186/s12917-020-02501-5 (PMC7398340; doi:10.1186/s12917-020-02501-5)
Supplement: Supplementary file 3 — Additional file 3. The sequences of the primers used in the individual polymerase chain reaction mixtures, the length of the product in base pairs, and the access number in the GenBank database. Detailed data regarding primers used in PCR. [file 12917_2020_2501_MOESM3_ESM.docx]

Additional file 3. The sequences of the primers used in the individual polymerase chain reaction mixtures, the length of the product in base pairs, and the access number in the GenBank database.

| **Mixture reaction*** | **Name of primers** | **Sequence of primers^#^** | **Amplicon (bp)** | **Access number/ reference** |
| --- | --- | --- | --- | --- |
| Mixture PCR I | *Pn1* | 5’ GGCAAATATGTCCCACGTGC 3’ | 580 | Z69258.1 |
|  | *Pn2* | 5’ GATCAGCATTCAGCTGTCCA 3’ |  |  |
| Mixture PCR II | Pn3 | 5’ GGAACAATGGACTCTGTTCG 3’ | 518 | Z69258.1 |
|  | Pn4 | 5’ GGGAATCGAAGGCAGGTCGT 3’ |  |  |
| Mixture ASA-PCR *H.contortus (1)* | Ph1 | 5’ GGAACGATGGACTCCTTTCG 3’ | NS 750 + S 550 | M76493.1 |
|  | Ph2 | 5’ GATCAGCATTCAGCTGTCCA 3’ |  |  |
|  | Ph4 | 5’ATACAGAGCTTCGTTGTCAATACAGA3’ |  |  |
| Mixture ASA-PCR *H.contortus (2)* | Ph1 | 5’ GGAACGATGGACTCCTTTCG 3’ | NS 750 + R 250 | M76493.1 |
|  | Ph2 | 5’ GATCAGCATTCAGCTGTCCA 3’ |  |  |
|  | Ph3 | 5’ CTGGTAGAGAACACCGATGAAACATA 3’ |  |  |
| Mixture ASA-PCR *T.colubriformis (1)* | Pc1 | 5’ GGAACAATGGATTCCGTTCG 3’ | NS 750 + S 550 | AH003221.2 |
|  | Pc2 | 5’ GGGAATCGGAGGCAAGTCGT 3’ |  |  |
|  | Pc3 | 5’ CTGGTAGAGAATACCGATGAAACATA 3’ |  |  |
| Mixture ASA-PCR *T.colubriformis (2)* | Pc1 | 5’ GGAACAATGGATTCCGTTCG 3’ | NS 750 + R 250 | AH003221.2 |
|  | Pc2 | 5’ GGGAATCGGAGGCAAGTCGT 3’ |  |  |
|  | Pc4 | 5’ATACAGAGCTTCGTTATCGATGCAGA 3’ |  |  |
| Mixture ASA-PCR *T.circumcincta* | Pt1 | 5’ GGAACAATGGACTCTGTTCG 3’ | NS 750 + S 550 + R 250 | Z69258.1 |
|  | Pt2 | 5’ GATCAGCATTCAGCTGTCCA 3’ |  |  |
|  | Pt3 | 5’ TTGGTAGAAAACACCGATGAAACATA 3’ |  |  |
|  | Pt4 | 5’ GTACAGAGCTTCATTATCGATGCAGA 3’ |  |  |

*Mixture reaction PCR I – the first PCR in nested PCR; Mixture reaction PCR II – the second PCR in nested PCR; ASA-PCR – allele-specific PCR; (1) – the first mixture with three single primers of a given species in ASA-PCR; (2) – the second mixture with three single primers of a given species in ASA-PCR; NS- non-specific primer; S – specific fragment; R – resistant fragment.

^#^Source of primers: Silvestre A, Humbert JF. A molecular tool for species identification and benzimidazole resistance diagnosis in larval communities of small ruminant parasites. Exp Parasitol. 2000;95:271-6.
