# Supplementary material for: The first report of multidrug resistance in gastrointestinal nematodes in goat population in Poland
Source: BMC Vet Res. 2020 Aug 3;16:270. doi: 10.1186/s12917-020-02501-5 (PMC7398340; doi:10.1186/s12917-020-02501-5)

Additional file 4. Original, full-length gel images presenting results of PCR for the length of restriction fragments of variant-1 of the β-tubulin gene indicating the resistance of *Trichostrongylus colubriformis* (A) and *Haemonchus contortus* (B) to benzimidazoles.


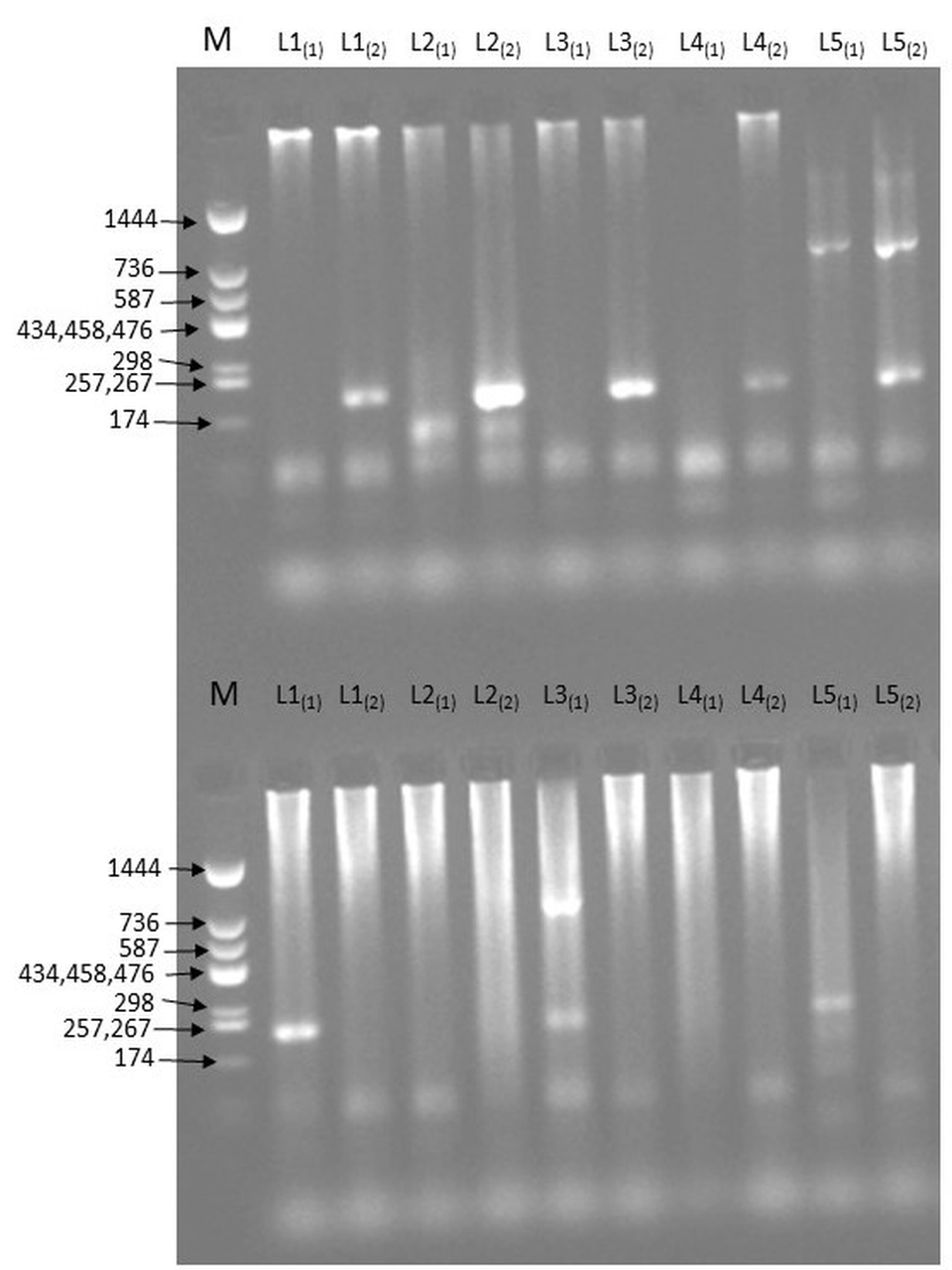

Supplement: Supplementary file 4 — Additional file 4. Original, full-length gel images presenting results of PCR for the length of restriction fragments of variant-1 of the β-tubulin gene indicating the resistance of Trichostrongylus colubriformis (A) and Haemonchus contortus (B) to benzimidazoles. Uncropped gels shown in Fig. 3 (A – Trichostrongylus colubriformis) and Fig. 4 (Haemonchus contortus – B). [file 12917_2020_2501_MOESM4_ESM.docx]
